# Evaluation of hand‐held sodium, potassium, calcium, and electrical conductivity meters for diagnosing subclinical mastitis and intramammary infection in dairy cattle

**DOI:** 10.1111/jvim.15550

**Published:** 2019-07-11

**Authors:** Sahar A. Kandeel, Ameer A. Megahed, Peter D. Constable

**Affiliations:** ^1^ Department of Veterinary Clinical Medicine, College of Veterinary Medicine University of Illinois at Urbana‐Champaign Champaign Illinois; ^2^ Department of Animal Medicine, Faculty of Veterinary Medicine Benha University Kalyobiya Egypt

**Keywords:** ion‐selective potentiometry, likelihood ratio, mastitis, somatic cell count

## Abstract

**Background:**

Subclinical mastitis (SCM) and intramammary infection (IMI) increase the sodium (Na) concentration and electrical conductivity (EC) and decrease the potassium (K) and calcium (Ca) concentrations in glandular secretions of lactating dairy cattle.

**Hypothesis:**

Low‐cost portable Na, K, Ca, and EC meters are clinically useful cow‐side tests for diagnosing SCM and IMI.

**Animals:**

One hundred fifteen dairy cows at dry off and 92 cows within 4‐7 days postcalving.

**Methods:**

Quarter foremilk samples were obtained and the somatic cell count (SCC) was measured using a DeLaval cell counter with SCM defined as SCC ≥ 200 000 cells/mL. Microbiological culture of foremilk samples was used to diagnose IMI. Cisternal milk Na, K, and Ca concentrations and EC were measured using portable ion‐selective meters. Logistic regression was used to determine the area under the receiver operating characteristic curve (AUC) and the optimal cut point was determined using Youden's index. Diagnostic test performance was evaluated by comparing the AUC and calculating the sensitivity, specificity, and positive likelihood ratio (+LR) at the optimal cut point for SCM and IMI.

**Results:**

Diagnostic test performance was much better when the meters were used to diagnose SCM as compared to IMI. Cisternal milk Na concentration provided the most accurate method for identifying quarters with SCM or IMI. However, AUC was <0.90 and +LR was <10 for all diagnostic test evaluations.

**Conclusions and Clinical Importance:**

Cisternal milk Na, K, and Ca concentrations and EC were not sufficiently predictive of SCM or IMI to be recommended as clinically useful diagnostic tests.

**Abbreviations**
+LR: positive likelihood ratio
κ: kappa coefficient
AUC: area under the curve
Ca: calcium
Cl: chloride
CMT: California mastitis test
DCC: DeLaval cell counter
EC: electrical conductivity
IMI: intramammary infection
K: potassium
MG: microbial growth
Na: sodium
ROC: receiver operating characteristic
SCC: somatic cell count
SCM: subclinical mastitis
Se: sensitivity
Sp: specificity

## 1 INTRODUCTION

Accurate identification of cows with subclinical mastitis (SCM) or intramammary infection (IMI) at dry off or early lactation is an important goal of mastitis control programs and a fundamental requirement for implementing selective dry cow treatment.^1^ Informative studies conducted 60 years ago documented that sodium (Na) concentrations increased and potassium (K) concentrations decreased in fat‐free milk samples from cows with mastitis, compared with healthy cows at a similar stage of lactation.^2^ The change in milk Na and K concentrations in mastitis is related to mastitis pathogen, severity of inflammation, decrease in lactose production, and milk fraction.^3^, ^4^, ^5^, ^6^, ^7^

The blood‐milk barrier is damaged in quarters with mastitis, causing the tight junctions between secretory cells to become leaky, thereby promoting the movement of extracellular fluid components, including Na and chloride (Cl), into the alveolus lumen where they mix with milk and increase the Na and Cl concentrations.^8^, ^9^ A concurrent decrease in alveolar milk K concentration occurs in response to the increased Na concentration to maintain constant milk osmolality.^10^, ^11^, ^12^ The laboratory determination of milk Na and K concentration was first introduced in 1972 as a method to detect tissue damage and increased permeability in quarters with SCM.^12^ Unfortunately, the inability to measure milk Na and K concentration rapidly and at low cost on farms has markedly impacted the widespread adoption of these indices to diagnose SCM.

Milk electrical conductivity (EC) is determined by the total concentration of cations and anions. Consequently, the net effect of increased milk Na and Cl concentrations, even in the presence of decreased milk K concentration, is an increase in milk EC.^13^, ^14^ The EC of bovine milk was first measured in 1943^12^ and several low‐cost portable EC instruments have been developed as a proxy for identifying quarters with SCM. However, milk EC is influenced by milk fat concentration, milk fraction, temperature, stage of lactation, and parity,^4^, ^5^, ^6^, ^15^, ^16^ decreasing the specificity (Sp) of EC in identifying SCM. Moreover, the predictive value of EC in diagnosing mastitis is considered inferior to that of the California mastitis test (CMT).^17^, ^18^

The LAQUAtwin meters (Horiba Ltd, Albany, New York) are hand‐held direct (ie, no dilution required) ion‐selective electrode meters that recently have become available for measuring Na, K, and Ca concentrations and EC in biological fluids. The low‐cost (approximately $250 US) and portability of the meters, as well as their ability to measure cation concentrations or EC in uncentrifuged milk samples, make them very attractive for on‐farm or cow‐side use. Studies conducted by our laboratory over the past 5 years have validated the accuracy of Na and K LAQUAtwin meters in measuring unbound Na and K concentrations in bovine cisternal milk.^19^, ^20^ The Ca LAQUAtwin meter has been used to measure ionized Ca concentration in bovine milk.^21^, ^22^ Based on these findings, we hypothesized that measuring cisternal milk Na and K concentrations using LAQUAtwin meters would provide accurate, low‐cost, and practical methods for diagnosing SCM and IMI in dairy cattle. Therefore, our objectives were to determine and compare the accuracy of LAQUAtwin portable EC, Na, K, and Ca meters for diagnosing SCM and IMI in dairy cattle.

## 2 MATERIALS AND METHODS

### 2.1 Samples

A prospective observational study was conducted at the University of Illinois Dairy Research Farm using a convenience sample of 115 cows sampled during the last week of lactation and 92 cows sampled 4 to 7 days postpartum over a period of 13 months between July 1, 2015 and July 31, 2016. A total of 130 animals calved during the course of the study; samples at freshening were not obtained from 38 of these animals because of workload constraints or the availability of analytical equipment that met calibration requirements. All methods were evaluated and approved by the University of Illinois Institutional Animal Care and Use Committee. The study reported here was part of a series of studies investigating the diagnosis of SCM and IMI in lactating dairy cattle.^23^, ^24^, ^25^, ^26^, ^27^ The management, housing, and feeding of study animals have been published elsewhere.^24^

A farm visit was performed at least once a week to collect foremilk (cisternal) samples from each quarter separately in the same week of drying off between 12:00 and 16:00 and at 4 to 7 days postpartum (to exclude colostral milk) between 12:00 and 14:00. Samples therefore were collected 8 to 12 hours after the previous milking at dry off and 7 to 9 hours after the previous milking at freshening. Somatic cell counts at these time intervals after the previous milking are similar to those obtained immediately before milking, which is the recommended time for accurate interpretation of somatic cell count (SCC) values.^28^ Cows at dry off were moved to a shaded area of the free stall and secured with the aid of a halter. Disposable gloves were applied by the investigators and the teat end of each quarter was cleaned with alcohol 70%.^29^ Twenty milliliters of milk samples were collected separately by hand stripping after discarding the first 3 squirts within 50 seconds of first touching any teat. This ensured that samples reflected cisternal milk^5^, ^6^, ^30^ and permitted the investigators to verify that the sampled cows did not have clinical mastitis. Samples from quarters with clinical mastitis detected by farm staff were obtained in a similar manner. Clinical mastitis was defined as a visibly abnormal glandular secretion when foremilk samples were examined before milking or abnormal udder changes, such as swelling, erythema, and pain, were evident. Samples were stored after collection in iced water in an insulated container and transported to laboratories at the dairy and College of Veterinary Medicine for analysis.

### 2.2 Analysis of cisternal milk

The reference method for determining SCC in our study was the DCC (DeLaval cell counter, DeLaval International AB, Tumba, Sweden), which is a portable automatic cell counter with a reported measurement range of 10 000 to 4 000 000 cells/mL and repeatability (coefficient of variation [CV]) of 12% at 100 000 cells/mL and 8% at 400 000 cells/mL. The DCC method has been validated for bovine milk at 4 and 37°C against the Fossomatic and direct microscopic methods^30^, ^31^, ^32^, ^33^ and was used as reference method in our study.

Samples were measured using the DCC at the University of Illinois Dairy Research Farm as recommended by the manufacturer. Approximately 1 μL of foremilk was drawn into the single‐use cassette and inserted into the DCC analyzer. The result was shown on the display after 45 seconds and indicated the number of cells/μL of milk; this number was multiplied by 1000 to provide cells/mL of milk.

**Figure 1:**
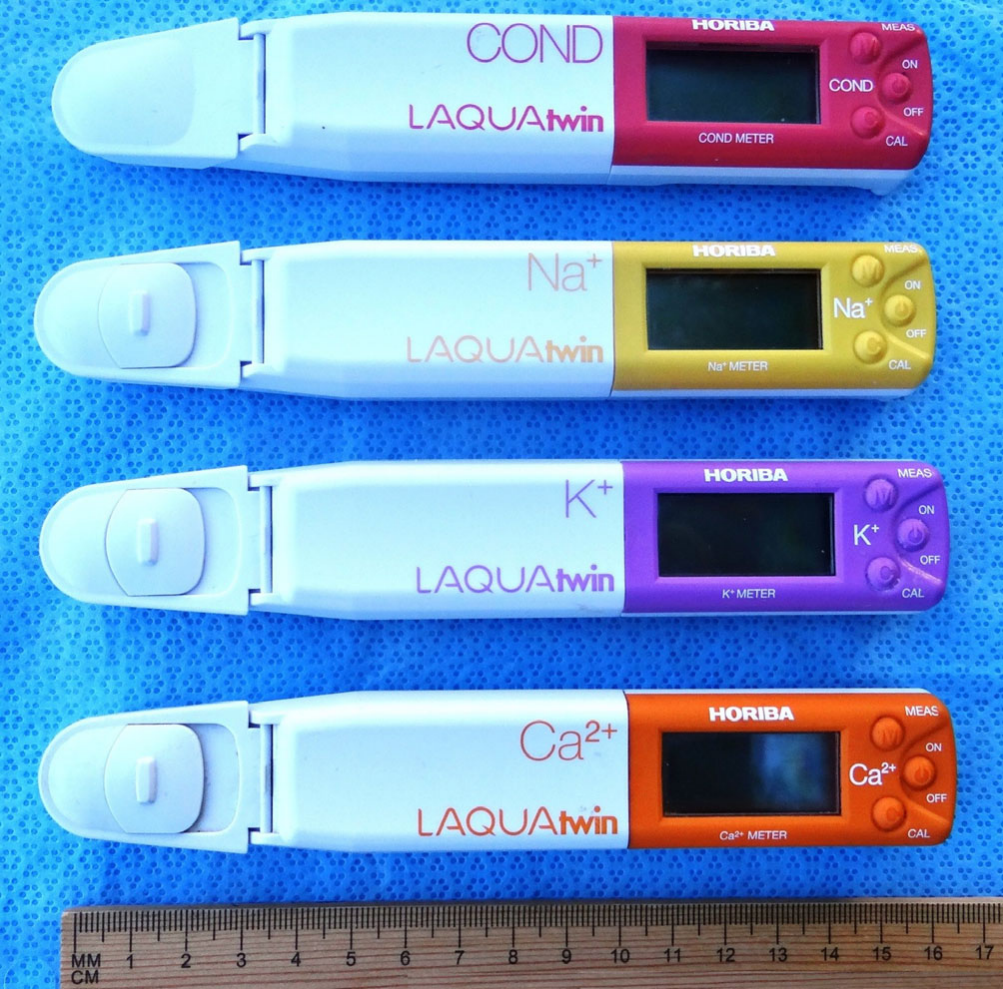
Horiba LAQUAtwin hand‐held direct ion selective electrode (ISE) meters for measuring electrical conductivity and the Na, K, and Ca concentrations in biological fluids

Milk EC and electrolyte concentrations were measured on quarter milk samples within 4 hours of collection; the delay was related to workload constraints during the course of the study. Milk samples were transported back to the laboratory and placed in water bath at 37°C for at least 30 minutes to approximate milk temperature when used as a cow‐side or on‐farm test. Portable meters were used for measuring milk EC (Cond LAQUAtwin meter “B‐771,” Horiba Ltd), and Na (Na LAQUAtwin meter “B‐722,” Horiba Ltd), K (K LAQUAtwin meter “B‐731,” Horiba Ltd), and Ca (Ca LAQUAtwin meter “B‐751,” Horiba Ltd) concentrations in quarter foremilk samples according to the manufacturer's recommendations. These hand‐held meters were suitable for cow‐side or on‐farm use (Figure 1). Electrical conductivity was calibrated at least once a day using a calibration solution of 1.41 mS/cm. The Na, K, and Ca meters were calibrated at least once a day using a 2‐point method and 2000 and 150 ppm standard solutions according to the manufacturer's recommendations. The measurement mode was accessed after calibration, the protective cover was opened, and the recommended amount of well‐mixed milk sample was transferred onto the flat sensor using a disposable pipette, and the protective cover of the meter was closed immediately. Measurements were recorded when the displayed value was stable, which was usually within 1 minute of sample placement onto the sensor. The response time of the meter depends on different sample factors, including ionic strength, protein content, and lipid content.^34^ After measurement of each sample, the sensor was cleaned by rinsing with distilled water and removing of any residual water using a tissue (Kimwipes Delicate Task Wipers, Kimberly Clark, Wisconsin). This ensured that there was no moisture on the sensor and the reading returned to “0” before measuring the next sample.

Milk pH was measured using glass‐electrodes pH meter (PICCOLO plus glass bulb single junction pH meter, Hanna Instruments, Woonsocket, Rhode Island) as described elsewhere.^26^ Briefly, the meter was calibrated using pH = 7.0 and 4.0 buffer solutions. After calibration, the electrode was dipped in the milk sample without exceeding the maximum immersion level. The pH of the sample was recorded after the reading had stabilized.

### 2.3 Microbiologic culture of cisternal milk

Milk culturing was performed based on National Mastitis Council recommendations^29^ using blood agar and MacConkey plates (trypticase soy agar with 5% sheep blood agar, MacConkey plates; Remel, Lenexa, Kansas). The plates were divided into 2 halves and labeled with cow ID and quarter. A 0.1 mL aliquot (100 μL) of each quarter milk sample was placed on one‐half of the blood agar and MacConkey plates separately and then streaked using a sterile wire loop in a manner that permitted growth of isolated bacterial colonies. Plates were incubated in an inverted position at 37°C for 48 hours and the microbial growth (MG) and colony type recorded. Isolated pathogens were identified by colony morphology, hemolysis pattern, biochemical tests including catalase and coagulase test, Gram‐staining reaction, and cell morphology according to National Mastitis Council guidelines.^29^ The microbiological isolates are reported elsewhere.^25^ Coagulase negative staphylococci were the most common isolate, being present in 12.4% of quarters at dry off and 8.2% of quarters at freshening.

### 2.4 Statistical analysis

Statistical software programs (SAS 9.4, SAS Inc, Cary, North Carolina; MedCalc Software bvba, Ostend, Belgium) were used for all analyses and *P* < .05 was considered significant. A minimum sample size of 300 quarters at dry off and freshening was identified as a goal based on what was logistically possible because of time and resource constraints balanced against the need for an adequate number of quarters to satisfactorily evaluate the diagnostic test performance of a new test for SCM or IMI. The measured ionized Ca concentration (*c*Ca^2+^_m_) was corrected for milk pH using the slope value ([Δ*c*Ca^2+^] /ΔpH = 2.714) from an equation developed for cow's milk at 37°C relating *c*Ca^2+^ to measured pH (pH_m_)^35^ and standardized to the experimentally determined median pH value for cisternal milk from healthy uninfected quarters at dry off at 37°C (6.60).^26^ The pH‐corrected equation for ionized Ca concentration (Ca_pH = 6.60_) therefore was: *c*Ca^2+^_pH = 6.60_ = *c*Ca^2+^_m_ + 2.71 × (pH_m_ − 6.60).

The least squares mean cisternal milk EC, ionized Na, K, and Ca concentrations, and the 95% confidence interval (CI) for the same 4 variables in healthy quarters (SCC < 100 000 cells/mL)^17^ were determined at dry off and freshening using mixed models analysis (PROC MIXED), with cow as a random effect and quarter nested within cow. Spearman's correlation coefficients (PROC CORR) were calculated to characterize the association between milk SCC and EC, as well as Na and K concentrations and *c*Ca^2+^_pH = 6.60_ measured by Horiba meters on quarter milk samples.

Subclinical mastitis was defined on a quarter basis as SCC ≥ 200 000 cell/mL because it is the most frequently used method and cutoff for diagnosing SCM, providing maximum sensitivity (Se) and Sp and minimal diagnostic error.^36^, ^37^ Microbial growth was defined as >10 colony‐forming units (cfu)/mL, equivalent to the isolation of a single colony from the 100 μL inoculum used in the study. We elected not to use a higher cut point of 100 cfu/mL^37^ in order to include infected quarters with low pathogen shedding or mild inflammatory response (eg, *Corynebacterium* spp. infection). Intramammary infection was defined on a quarter basis as the presence of MG and SCC ≥ 100 000 cells/mL; our definition for IMI therefore reflects the isolation of a pathogen from glandular secretion with evidence of inflammation. The SCC cut point for our IMI definition was selected because quarter SCC < 100 000 cells/mL reflects an internationally accepted definition of udder quarter health.^17^, ^38^

Binary logistic regression (PROC LOGISTIC) was used to characterize the relationship between SCM (1, SCC ≥ 200 000 cells/mL; 0, SCC < 200 000 cells/mL) or IMI (1, presence of both SCM and MG; 0, other), and milk EC, as well as Na and K concentrations and *c*Ca^2+^_pH = 6.60_ measured by the Horiba meters at dry off and freshening. Logistic regression was conducted on a quarter basis without consideration of cow effect reflecting interdependence of quarters. This analytical approach was selected because the practical implementation of selective dry cow antimicrobial administration should be based on the results of diagnostic testing of quarter samples without adjustment for cow factors.

Receiver operating characteristic (ROC) curves were constructed for each logistic regression model and the area under ROC curve (AUC) and 95% Wald confidence limits for the AUC were calculated. The AUC provides a global index of diagnostic test performance and a Wald *P*‐value of <.05 indicates that the test result is significantly better than a chance result. The AUC values for different predictors were compared using a nonparametric approach.^39^ Values for the AUC of ROC curve >0.9 typically indicate a highly accurate diagnostic test, whereas AUC values of 0.7 to 0.9 indicate moderate accuracy, 0.5 to 0.7 low accuracy, and 0.5 represents a chance result.^40^ The adequacy of the logistic regression model fit was evaluated using plots of deviance influence statistics against the predicted values.^41^ Sensitivity and Sp were calculated at the optimal cut point of each ROC that was determined using the Youden index (the cut point where the following expression has its maximum value: Se + Sp − 1). This analytical method equally weights the values of Se and Sp. The positive likelihood ratio (+LR) was calculated as: +LR Se/(1 − Sp); +LR values >10 indicate that a positive test is good at ruling in the diagnosis such as SCM or IMI.^42^ The kappa coefficient (κ, PROC FREQ) was calculated using the optimal cut point of the ROC to characterize the level of agreement between milk EC, and Na, K, and Ca concentration, measured by Horiba meters and the 2 reference methods (SCM and IMI). Values for κ < 0.2 indicate poor agreement, whereas 0.2 < κ < 0.4 indicates fair agreement, 0.4 < κ < 0.6 indicates moderate agreement, 0.6 < κ < 0.8 reflects good agreement, and κ > 0.8 indicates excellent agreement.^43^

## 3 RESULTS

### 3.1 Samples

Quarter milk samples were obtained at dry off from 115 cattle, comprising 102 Holstein Friesian, 9 Jersey, 2 Ayrshire, 1 Brown Swiss, and 1 Milking Shorthorn. The median SCC at dry off (459 quarter samples, with 1 cow having 1 blind quarter) was 356 000 cells/mL. Quarter milk samples were obtained at freshening from 92 cattle, comprising 81 Holstein Friesian, 8 Jersey, 1 Ayrshire, 1 Brown Swiss, and 1 Milking Shorthorn. The median SCC at freshening (364 quarter samples, with 4 cows having 1 blind quarter) was 113 000 cells/mL.

Of the 459 quarters sampled at dry off, 14% (n = 63) were considered healthy (SCC < 100 000 cells/mL), 68% (n = 312) had SCM, and 25% (n = 113) had IMI. The percentages summed to >100% because quarters could have both SCM and IMI. Of the 364 quarters sampled at freshening, 46% (n = 166) were considered healthy, 33% (n = 120) had SCM, and 17% (n = 61) had IMI. The percentages summed to <100% because some quarters had a SCC ≥ 100 000 cells/mL but <200 000 cells/mL with no MG.

**Figure 2:**
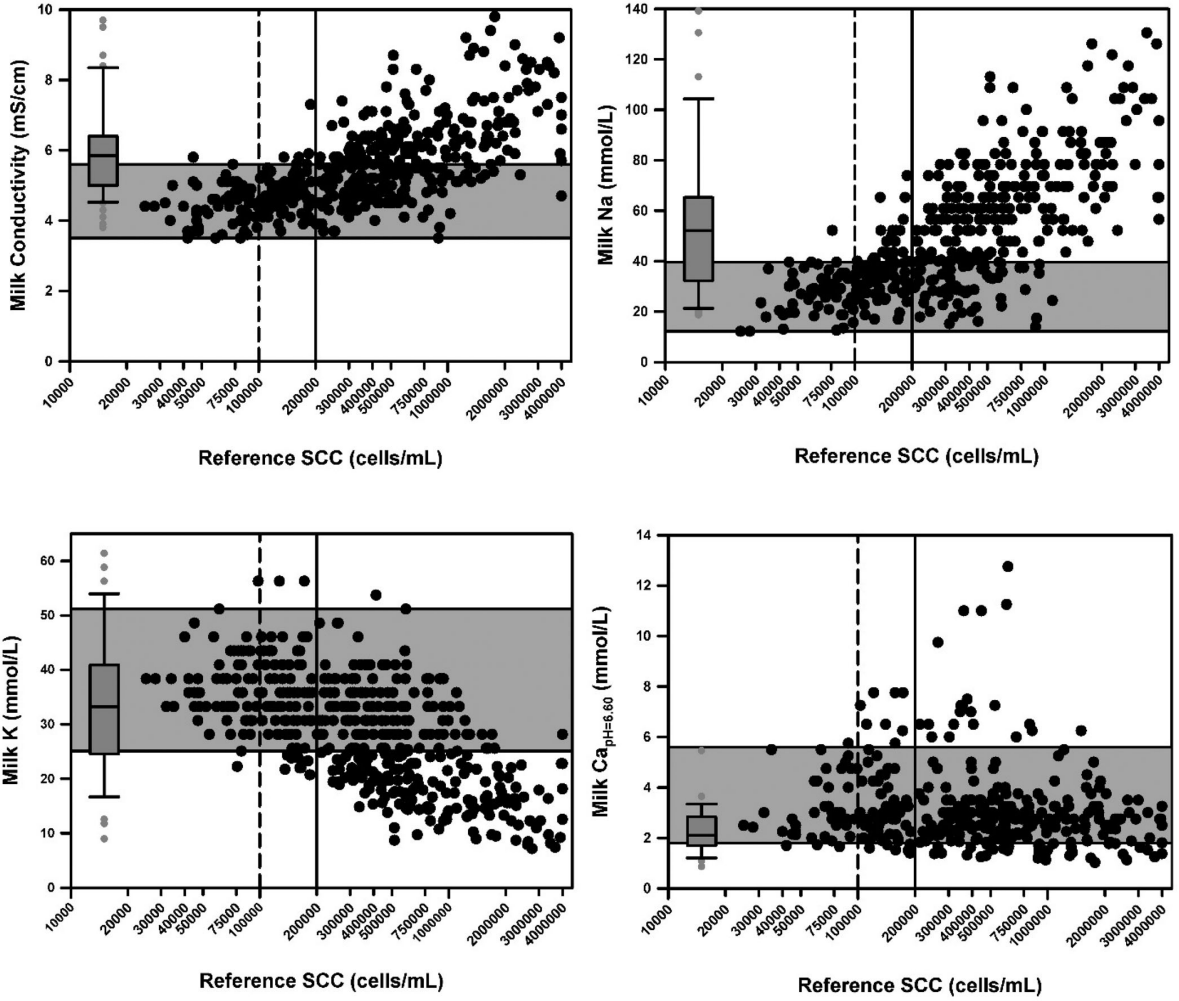
Scatterplots of the relationship between quarter milk electrical conductivity, as well as the Na, K, and Ca concentrations, and quarter somatic cell count (SCC) for 115 dairy cows at dry off. The dashed vertical line represents the current consensus limit for the SCC of healthy quarters (<100 000 cells/mL). The solid vertical line represents the definition used in this study for diagnosing subclinical mastitis (≥200 000 cells/mL). The horizontal shaded area represents the 95% confidence interval (CI) for the value in healthy quarters (SCC <100 000 cells/mL) at dry off. The box plot represents the median (middle line), interquartile range (ends of the shaded rectangle), 95% CI (whiskers), and values outside the 95% CI (small gray circles) for 74 quarters from 49 lactating cows with clinical mastitis. The median day in milk was 104 days when the samples were obtained from cows with clinical mastitis (range, 3‐416 days)

**Figure 3:**
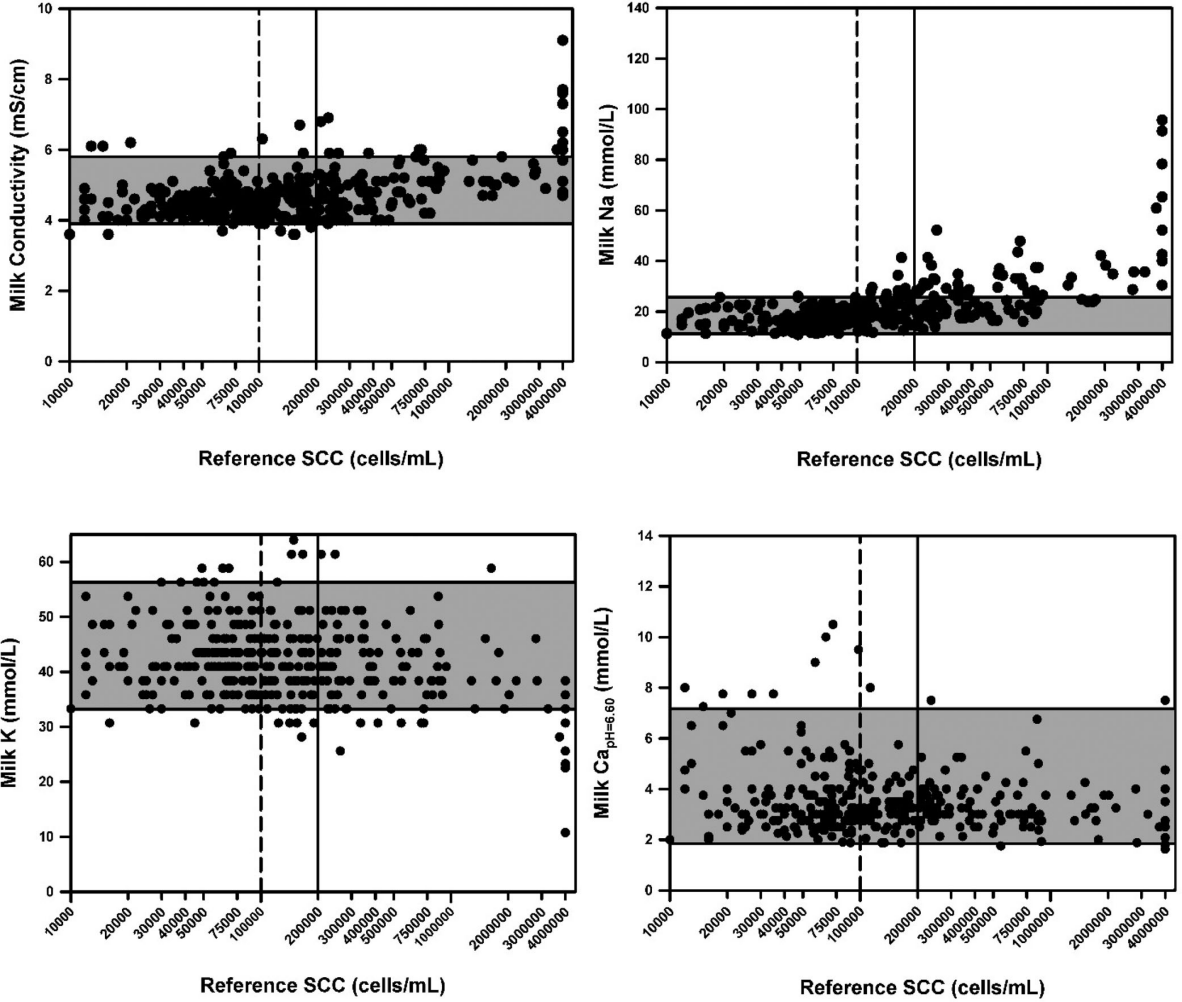
Scatterplots of the relationship between quarter milk electrical conductivity, as well as the Na, K, and Ca concentrations, and quarter somatic cell count (SCC) for 92 dairy cows at 4‐7 days after calving. The dashed vertical line represents the current consensus limit for SCC of healthy quarters (<100 000 cells/mL). The solid vertical line represents the definition used in this study for diagnosing subclinical mastitis (≥200 000 cells/mL). The horizontal shaded area represents the 95% confidence interval for the value in healthy quarters (SCC <100 000 cells/mL) at freshening

Scatterplots of the relationship of cisternal milk EC, as well as cisternal milk Na and K concentrations, and *c*Ca^2+^_pH = 6.60_ and SCC are provided at dry off (Figure 2) and freshening (Figure 3). Spearman correlation coefficients between variables of interest at dry off and freshening are summarized in Table 1. The correlation coefficients for the sum of Na and K concentrations, or the Na to K ratio, and the SCC were lower than that for the Na concentration at dry off.

**Table 1:** Spearman correlation coefficients among variables of interest for 115 dairy cows (1 blind quarter) at dry off and 92 cows (4 blind quarters) at freshening. The information in parentheses is the number of quarters used for comparison and the corresponding *P* value

Variable	Delaval SCC	EC	Na	K	Na + K	Na to K	Ca^2+^_pH = 6.60_
*Dry off*							
SCC	+1.00	+0.67 (n = 459, *P* < .001)	+0.72 (n = 416, *P* < .001)	−0.65 (n = 459, *P* < .001)	+0.67 (n = 416, *P* < .001)	+0.71 (n = 416, *P* < .001)	−0.04 (n = 364, *P* = .44)
EC		+1.00	+0.86 (n = 416, *P* < .001)	−0.71 (n = 459, *P* < .001)	+0.86 (n = 416, *P* < .001)	+0.81 (n = 416, *P* < .001)	−0.04 (n = 364, *P* = .40)
Na			+1.00	−0.89 (n = 416, *P* < .001)	+0.92 (n = 416, *P* < .001)	+0.98 (n = 416, *P* < .001)	+0.07 (n = 321, *P* = .21)
K				+1.00	−0.66 (n = 416, *P* < .001)	−0.96 (n = 416, *P* < .001)	−0.12 (n = 364, *P* = .02)
Na + K					+1.00	+0.83 (n = 416, *P* < .001)	+0.01 (n = 321, *P* = .82)
Na to K						+1.00	+0.10 (n = 321, *P* = .08)
*Freshening*							
SCC	+1.00	+0.40 (n = 364, *P* < .001)	+0.60 (n = 321, *P* < .001)	−0.23 (n = 345, *P* < .001)	+0.31 (n = 321, *P* < .001)	+0.61 (n = 321, *P* < .001)	−0.01 (n = 333, *P* = .86)
EC		+1.00	+0.50 (n = 321, *P* < .001)	+0.16 (n = 364, *P* = .001)	+0.62 (n = 321, *P* < .001)	+0.34 (n = 321, *P* < .001)	+0.23 (n = 333, *P* < .001)
Na			+1.00	−0.20 (n = 348, *P* < .001)	+0.66 (n = 321, *P* < .001)	+0.88 (n = 321, *P* < .001)	+0.35 (n = 320, *P* < .001)
K				+1.00	+0.54 (n = 321, *P* < .001)	−0.56 (n = 321, *P* < .001)	+0.04 (n = 333, *P* = .39)
Na + K					+1.00	+0.26 (n = 321, *P* < .001)	0.33 (n = 293, *P* < .001)
Na to K						+1.00	0.18 (n = 293, *P* = .002)

Abbreviations: Ca^2+^_pH = 6.60_, milk ionized calcium concentration corrected to a standard milk pH of 6.60; EC, electrical conductivity, K, milk potassium concentration; Na, milk sodium concentration; SCC, somatic cell concentration.

The median and 2.5%‐97.5% CI for milk EC, Na and K concentrations, and *c*Ca^2+^_pH = 6.60_ in healthy quarters at dry off were 4.5 (CI, 3.5‐5.6) mS/cm, 28 (CI, 12‐40) mmol/L, 36 (CI, 25‐51) mmol/L, and 3.0 (CI, 1.8‐5.6) mmol/L, respectively (Figure 2). For comparison, the median and 2.5%‐97.5% CI for milk EC, Na and K concentrations, and *c*Ca^2+^_pH = 6.60_ in healthy quarters at freshening were 4.5 (CI, 3.9‐5.8) mS/cm, 17 (CI, 11‐26) mmol/L, 44 (CI, 33‐56) mmol/L, and 2.9 (CI, 1.8‐7.2) mmol/L, respectively (Figure 3). Higher values and greater variability in quarter *c*Ca^2+^_pH = 6.60_ were identified with milk pH < 6.80 (data not shown).

### 3.2 Subclinical mastitis

Values for the optimal cut point, AUC, Se, Sp, +LR, and κ for diagnosing SCM based on the results of logistic regression analysis are presented in Table 2. Logistic regression analysis indicated that the AUC for milk Na concentration was not different (*P* = .06) from that for EC at dry off but was greater (*P* = .01) than that for EC at freshening. The AUC for milk K concentration was lower (*P* = .02) than that for EC at freshening. The AUC for milk Na concentration for diagnosing SCM was greater than that for milk K concentration at dry off (*P* = .004) and freshening (*P* < .001). The AUC for *c*Ca^2+^_pH = 6.60_ at dry off and freshening were lower than that of EC, Na concentration, and K concentration (*P* < .001). Furthermore, the Wald *P* values for AUC for *c*Ca^2+^_pH = 6.60_ at dry off (*P* = .21) and freshening (*P* = .66) indicated that ionized Ca concentration was not predictive of SCM. The κ values indicated moderate agreement among cisternal milk EC > 5.2 mS/cm, Na concentration > 39 mmol/L, and K concentration <29 mmol/L as diagnostic tests for SCM at dry off, and moderate agreement between milk EC > 4.9 mS/cm and Na concentration >24 mmol/L as diagnostic tests for SCM at freshening.

**Table 2:** Summary of the results of logistic regression analysis of the ability of electrical conductivity, and sodium, potassium, and calcium concentrations measured by ion‐selective potentiometry, to predict subclinical mastitis and intramammary infection in quarter milk samples obtained from dairy cows at dry off (n = 115) and freshening (n = 92). Subclinical mastitis was defined as a cisternal milk somatic cell count ≥200 000 cells/mL. Intramammary infection was defined as isolation of a microbiological agent ≥10 cfu/mL from an aseptically collected cisternal milk sample and a cisternal milk somatic cell count ≥100 000 cells/mL

	n	Optimal cut point	AUC	Se	Sp	+LR	κ
**Subclinical mastitis**							
*Dry off*							
Conductivity (mS/cm)	459	>5.2	0.83^ab^ (0.79‐0.87)	0.66 (0.60‐0.71)	0.88 (0.82‐0.93)	5.7 (3.6‐9.0)	0.47 (0.39‐0.54)
Sodium (mmol/L)	416	>39	0.86^a^ (0.82‐0.89)	0.81 (0.76‐0.85)	0.78 (0.70‐0.85)	3.7 (2.7‐5.2)	0.56 (0.48‐0.65)
Potassium (mmol/L)	459	<29	0.82^b^ (0.78‐0.86)	0.68 (0.63‐0.73)	0.82 (0.75‐0.88)	3.9 (2.7‐5.5)	0.45 (0.37‐0.53)
Calcium_pH = 6.60_ (mmol/L)	364	ND	0.52^c^ (0.46‐0.59)	ND	ND	ND	ND
*Freshening*							
Conductivity (mS/cm)	364	>4.9	0.72^a^ (0.66‐0.78)	0.53 (0.43‐0.62)	0.86 (0.81‐0.90)	3.7 (2.6‐5.2)	0.40 (0.30‐0.50)
Sodium (mmol/L)	321	>24	0.81^b^ (0.76‐0.86)	0.56 (0.46‐0.65)	0.91 (0.86‐0.94)	5.9 (3.8‐9.3)	0.50 (0.39‐0.60)
Potassium (mmol/L)	345	<39	0.60^c^ (0.54‐0.67)	0.53 (0.43‐0.62)	0.65 (0.58‐0.71)	1.5 (1.2‐1.9)	0.16 (0.06‐0.27)
Calcium_pH = 6.60_ (mmol/L)	333	ND	0.48^d^ (0.42‐0.54)	ND	ND	ND	ND
**Intramammary infection**							
*Dry off*							
Conductivity (mS/cm)	459	>5.0	0.58^a^ (0.52‐0.64)	0.70 (0.61‐0.78)	0.47 (0.41‐0.52)	1.3 (1.1‐1.5)	0.11 (0.04‐0.18)
Sodium (mmol/L)	416	>33	0.60^a^ (0.54‐0.66)	0.86 (0.78‐0.92)	0.32 (0.27‐0.38)	1.3 (1.1‐1.4)	0.12 (0.06‐0.17)
Potassium (mmol/L)	459	<23	0.59^a^ (0.53‐0.65)	0.44 (0.35‐0.54)	0.71 (0.66‐0.76)	1.5 (1.2‐2.0)	0.14 (0.04‐0.23)
Calcium_pH = 6.60_ (mmol/L)	364	ND	0.55^a^ (0.48‐0.62)	ND	ND	ND	ND
*Freshening*							
Conductivity (mS/cm)	364	>4.7	0.69^a^ (0.61‐0.76)	0.67 (0.54‐0.79)	0.64 (0.58‐0.69)	1.9 (1.5‐2.3)	0.19 (0.11‐0.28)
Sodium (mmol/L)	321	>24	0.73^a^ (0.65‐0.81)	0.60 (0.45‐0.73)	0.78 (0.73‐0.83)	2.8 (2.0‐3.8)	0.30 (0.18‐0.41)
Potassium (mmol/L)	345	<39	0.59^ab^ (0.51‐0.68)	0.52 (0.38‐0.66)	0.61 (0.55‐0.67)	1.3 (1.0‐1.8)	0.08 (0.00‐0.17)
Calcium_pH = 6.60_ (mmol/L)	333	ND	0.51^bc^ (0.43‐0.59)	ND	ND	ND	ND

*Note*: AUC values with different superscripts at dry off or freshening are significantly different.

Abbreviations: +LR, positive likelihood ratio; κ, kappa coefficient; AUC, area under the response characteristic curve; n, number of quarters used in the analysis; ND, not determined; Se, sensitivity; Sp, specificity.

### 3.3 Intramammary infection

Values for the optimal cut point, AUC, Se, Sp, +LR, and κ for diagnosing IMI based on the results of logistic regression analysis are presented in Table 2. Logistic regression analysis indicated that the AUC for milk Na concentration was greater than that for milk K concentration at freshening (*P* = .02). The κ values indicated poor agreement for cisternal milk EC >5.0 mS/cm, Na concentration >33 mmol/L, and K concentration <23 mmol/L as diagnostic tests for IMI at dry off, fair agreement for cisternal Na concentration >24 mmol/L as a diagnostic test for IMI at freshening, and poor agreement between cisternal milk EC >4.7 mS/cm and K concentration <39 mmol/L as diagnostic tests for IMI at freshening.

### 3.4 Clinical mastitis

Clinical mastitis was identified throughout lactation at milking in 93 quarters from 54 cows. Analysis was confined to 74 quarters from 49 cows that were culture positive on routine microbiological culture. The median day in milk was 104 days when the samples were obtained (range, 3‐416 days). The primary isolates in the 74 quarters were Gram‐negative rods (35 quarters, predominantly *Klebsiella pneumoniae* and *Serratia marcescens*) and Gram‐positive cocci (12 quarters, predominantly *Streptococcus dysgalactiae*). Ten quarters had *Prototheca* spp. isolated, 8 quarters had coagulase‐negative staphylococci isolated, 4 quarters had yeast, 3 quarters had a Gram‐positive rod isolated (all *Corynebacterium* spp.), and 2 were considered contaminated. The median milk EC, Na and K concentrations, and *c*Ca^2+^_pH = 6.60_ in quarters with clinical mastitis were 5.8 mS/cm, 52 mmol/L, 33 mmol/L, and 2.0 mmol/L, respectively (Figure 2).

## 4 DISCUSSION

We compared the clinical utility of using portable low‐cost meters that measure milk EC and Na, K, and Ca concentrations as on‐farm or cow‐side tests for diagnosing SCM and IMI at dry off and freshening. We considered the quarter as the unit of analysis, and assumed that test Se and Sp were of equal importance. On this basis, the AUC, +LR, and κ coefficient provide clinically useful indices of overall test performance of the methods under evaluation. Based on our finding that AUC < 0.90, +LR < 10, and κ < 0.60 for all diagnostic test evaluations, our primary findings were that cisternal milk Na, K, and Ca concentrations and EC are not sufficiently predictive of the presence of SCM or IMI at dry off or freshening to be recommended as clinically useful diagnostic tests.

A positive relationship between milk EC and SCC has been demonstrated in many studies,^13^, ^15^ including our study (*r*_s_ = +0.67). This association does not necessarily mean EC is a useful diagnostic test, because such tests should have sufficiently high Se and Sp and therefore +LR >10 to provide clinically useful predictions of the presence of SCM. Our results indicated that the median EC for milk from healthy quarters at 37°C was 4.5 mS/cm, and that EC provided a moderately accurate method for diagnosing SCM at an optimal cut point of >5.2 mS/cm at dry off (moderate agreement based on κ = 0.47) and >4.9 mS/cm at freshening (moderate agreement based on κ = 0.40). Our findings agreed with another report that the mean milk EC of healthy cows was 4.9 mS/cm at temperatures approximating rectal and milk temperature, whereas mean milk EC increased to 5.4 and 6.4 mS/cm in cows with SCM or clinical mastitis, respectively.^14^ Mean cisternal milk EC in quarters with SCM was increased to temperature‐corrected values^15^ of 7.8 or 8.0 mS/cm at 37°C (SCC, 776 000‐1 413 000 cells/mL).^4^ Variation in the optimal EC cut off for diagnosing SCM between studies is related to fat concentration, proportion of milk solids, and the milk fraction.^15^, ^16^, ^44^ A review indicated that breed can affect EC,^13^ and EC varies with genotype,^45^ but the results of the only study we were able to identify that specifically examined the effect of breed indicated similar EC values for Black and White, Simmental, and Brown Swiss breeds.^46^ An effect of breed on the test performance of EC may be difficult to confirm because EC is mainly influenced by the severity of inflammation and the milk fat percentage, with the latter variable largely dependent on the milk fraction.^3^, ^4^ Milk fat percentage also changes in inappetent cows and cows with ruminal acidosis,^47^ fatty liver, ketosis, and displaced abomasum.^48^ In summary, an increase in milk EC is not specific for an increase in SCC, and a 1998 meta‐analysis concluded that “the published information is too varied to justify a claim that mastitis, especially subclinical mastitis, can be detected by means of EC measurements in milk.”^13^

Sodium is a plasma electrolyte that passes into milk at low levels of cellular injury,^4^ suggesting that milk Na concentration provides a sensitive indicator of the magnitude of tissue injury in quarters with SCM or clinical mastitis.^16^ We found that the portable Na meter provided a moderately accurate method for diagnosing SCM at an optimal cut point of >39 mmol/L at dry off (moderate agreement based on κ = 0.56) and >24 mmol/L at freshening (moderate agreement based on κ = 0.50). We also found that the mean Na concentration of cisternal milk from healthy quarters was 28 and 17 mmol/L at dry off and freshening, respectively, and that quarter milk Na concentration was increased in cows with clinical mastitis. The Na concentration in cisternal milk from uninfected quarters measured by ion‐elective potentiometry was reported to be 23 mmol/L at week 4 of lactation^3^ and 19‐22 mmol/L (stage of lactation not reported).^4^ Mean milk cisternal Na concentration increased by 7‐8 to 26‐30 mmol/L in quarters with SCM (SCC, 776 000‐1 413 000 cells/mL).^4^ A marked increase in the mean fat‐free (skimmed) milk Na concentration occurs in late lactation from 30 mmol/L in early lactation and 25 mmol/L in mid‐lactation to 49 mmol/L in late lactation.^2^ A decrease in lactose production in late lactation is the most likely reason for the increased milk Na concentration at dry off.

Potassium is the predominant mineral in milk from healthy quarters on a mmol/L basis.^2^, ^7^ Our results indicated that the mean K concentration of cisternal milk from healthy quarters was 36 and 44 mmol/L at dry off and freshening, respectively. For comparison, the mean K concentration in cisternal milk from uninfected quarters measured by ion‐selective potentiometry was reported to be 33 mmol/L at week 4 of lactation^3^ and 35‐36 mmol/L (lactation stage not reported).^4^ We found that the portable K meter provided a moderately useful to poor method for diagnosing SCM at an optimal cut point of <29 mmol/L at dry off (κ = 0.45) or <39 mmol/L (κ = 0.16) at freshening. Mean cisternal milk K concentration, measured by ion‐selective potentiometry, decreased by 2‐3 to 32‐36 mmol/L in quarters with SCM (SCC, 776 000‐1 413 000 cells/mL),^4^ and mean quarter milk K concentrations decreased to 36 and 28 mmol/L in *Staphylococcus aureus*‐infected quarters with SCC of 250 000‐500 000 and >750 000 cells/mL milk, respectively.^7^ However, the correlation coefficients for the SCC and the sum of Na and K concentrations, or the Na to K ratio, were lower than that for the Na concentration at dry off, suggesting that measuring both Na and K concentrations does not provide additional information at dry off.

Mammary secretory cells have pumps in their basolateral membranes that pump Na into the extracellular fluid and K into the cells, whereas Na and K are passively transported across the apical membrane of secretory cells into milk.^8^, ^17^, ^49^ The observed decrease in milk K concentration in quarters with SCM has been attributed to the need to maintain an isotonic milk osmolality, whereby the increase in milk Na concentration results in a proportional decrease in milk K concentration by moving K from milk to lymph through the paracellular pathway between damaged secretory cells, thereby decreasing milk K concentration.^49^ A small decrease in the mean fat‐free (skimmed) milk K concentration occurs in late lactation for unknown reasons, from 42 mmol/L in early lactation and 40 mmol/L in mid‐lactation to 27 mmol/L in late lactation.^2^ An increase in milk Na concentration because of decreased lactose production is the most likely reason for the decreased milk K concentration at dry off. In summary, the superior performance of milk Na concentration over milk K concentration in diagnosing SCM is consistent with our current understanding that milk K concentration decreases in response to an increase in milk Na concentration, and that the magnitude of the change in cisternal milk Na concentration is greater than that of cisternal K concentration.

Our results indicated that the cisternal milk ionized Ca concentration corrected to a standardized pH of 6.60 was not predictive of SCM. Milk pH increases with increased milk SCC,^26^, ^35^ resulting in a pH‐dependent decrease in the measured ionized Ca concentration.^35^ Cisternal milk ionized Ca concentration is also decreased in mastitis because of a decrease in total Ca concentration as a consequence of decreased casein synthesis and correspondingly lower milk casein concentration.^7^ Bovine milk from healthy quarters contains a total Ca concentration of approximately 30 mmol/L,^7^, ^35^ and ionized Ca concentration typically represents <10% of the total Ca concentration in bovine milk.^35^ The median pH‐corrected ionized Ca concentration for bovine milk in our study of 3.0 mmol/L at dry off therefore was consistent with previous findings. Ionized Ca concentrations >6 mmol/L were measured in approximately 10% of the quarter samples at dry off, and these all came from cisternal milk samples with measured pH < 6.80. The most likely reason for this finding was a pH‐dependent exchange of Ca, P, and citrate as well as other minerals between the aqueous phase of milk and the casein micelle.^35^, ^50^ A wide range of milk ionized Ca concentration in individual cows has been reported previously in the presence of much lower variability in milk total Ca concentration.^21^, ^35^ Causes for the cow‐to‐cow variability in ionized Ca concentration require further investigation, but this variability is likely to decrease the clinical utility of measuring ionized Ca concentration to identify quarters with SCM.

We elected not to report predictive values of a positive test (PV+) or a negative test (PV−) in our study. These 2 indices are influenced by the prevalence of disease in the sample population and consequently PV+ and PV− can only accurately summarize test performance when the study population is representative of the total population. In contrast, estimates for Se, Sp, and +LR are characteristics of the test itself and are not influenced by disease prevalence.

The major limitation of our study was that it was based on only 1 herd with the most common isolates being environmental mastitis pathogens. We sampled foremilk after removal of approximately 10 mL of milk but before alveolar milk could mix with cisternal milk.^30^ This sampling strategy was selected to minimize within cow variability in SCC as a consequence of all samples being collected within 50 seconds of first touching the udder and teat, and to reflect sampling practices on dairies where foremilk samples are most commonly collected for determination of IMI.^51^ Foremilk SCC ranging from 50 000 to 300 000 cells/mL has a similar SCC to cisternal and alveolar milk.^52^ On this basis, we believe that the SCC cut points of 200 000 cells/mL (SCM) and 100 000 cells/ml (IMI) used in this study approximated those employed in other studies where milk samples most likely reflected an admixture of cisternal and alveolar milk.^53^

The proportion of cattle of various dairy breeds in our study approximates that of the US dairy industry, and consequently our results should be generalizable to dairy cattle in the United States. However, a potential limitation of our study was that we ignored the effect of sample interdependence as a result of quarters being clustered within cows. As such, the reported CI for Se and Sp in our study underestimated the true CI obtained using random effects logistic regression analysis.^37^ However, use of random effects logistic regression in a much larger data set comprising 5344 quarter samples from 1342 cows in 90 herds did not identify a detectable change in the CI for Sp estimates.^37^ Moreover, because only minor differences in the Cis for Se estimates were identified in that study, the authors elected to present all CI as unconditional estimates for the sake of simplicity,^37^ similar to our study.

It is of interest to compare the results of the study reported here for diagnosing quarters with SCM to our findings in this study population^25^ that a CMT score of trace or higher had AUC = 0.95, Se = 0.95, Sp = 0.86, +LR = 6.7, and κ = 0.81 at dry off, and AUC = 0.88, Se = 0.79, Sp = 0.95, +LR = 15.3, and κ = 0.76 at freshening. Comparison of the diagnostic test performance of the CMT to the point‐of‐care tests evaluated in our study indicated that cisternal milk Na, K, and Ca concentrations and EC were not as accurate as CMT in diagnosing SCM at dry off and freshening. We therefore conclude that the diagnostic performance of the Horiba LAQUAtwin meters in measuring cisternal milk Na and K concentrations and EC, although statistically significant predictors of SCM and IMI in quarter milk samples obtained at dry off and freshening, was not sufficiently accurate to recommend these tests over the CMT as screening tests to identify quarters with SCM or IMI. Additional studies conducted in different cattle populations are indicated to confirm our results.

## CONFLICT OF INTEREST DECLARATION

Authors declare no conflict of interest.

## OFF‐LABEL ANTIMICROBIAL DECLARATION

Authors declare no off‐label use of antimicrobials.

## INSTITUTIONAL ANIMAL CARE AND USE COMMITTEE (IACUC)

The study had University of Illinois at Urbana IACUC approval number 15080.

## HUMAN ETHICS APPROVAL DECLARATION

Authors declare human ethics approval was not needed for this study.
